# Cost-effectiveness of seasonal influenza vaccination in WHO-defined high-risk populations in Bangladesh

**DOI:** 10.7189/jogh.14.04126

**Published:** 2024-07-19

**Authors:** Md Zakiul Hassan, Md Abdullah Al Jubayer Biswas, Tahmina Shirin, Mahmudur Rahman, Fahmida Chowdhury, Eduardo Azziz-Baumgartner, William W Davis, Mofakhar Hussain

**Affiliations:** 1 Program for Emerging Infections, Infectious Disease Division, International Centre for Diarrhoeal Disease Research, Bangladesh (icddr,b), Mohakhali, Dhaka, Bangladesh; 2 Pandemic Sciences Institute, Nuffield Department of Clinical Medicine, University of Oxford, Oxford, UK; 3 Institute of Epidemiology, Disease Control and Research (IEDCR), Dhaka, Bangladesh; 4 Global Health Development the Eastern Mediterranean Public Health Network (EMPHNET), Dhaka, Bangladesh; 5 Influenza Division, Centers for Disease Control and Prevention, Atlanta, Georgia, USA; 6 Institute of Health Policy, Management and Evaluation, University of Toronto, Canada

## Abstract

**Background:**

Bangladesh carries a substantial health and economic burden of seasonal influenza, particularly among the World Health Organization (WHO)-defined high-risk populations. We implemented a modelling study to determine the cost-effectiveness of influenza vaccination in each of five high-risk groups (pregnant women, children under five years of age, adults with underlying health conditions, older adults (≥60 years), and healthcare personnel) to inform policy decisions on risk group prioritisation for influenza vaccination in Bangladesh.

**Methods:**

We implemented a Markov decision-analytic model to estimate the impact of influenza vaccination for each target risk group. We obtained model inputs from hospital-based influenza surveillance data, unpublished surveys, and published literature (preferentially from studies in Bangladesh, followed by regional and global ones). We used quality-adjusted life years (QALY) as the health outcome of interest. We also estimated incremental cost-effectiveness ratios (ICERs) for each risk group by comparing the costs and QALY of vaccinating compared to not vaccinating each group, where the ICER represents the additional cost needed to achieve one year of additional QALY from a given intervention. We considered a willingness-to-pay threshold (ICER) of less than one gross domestic product (GDP) per capita as highly cost-effective and of one to three times GDP per capita as cost-effective (per WHO standard). For Bangladesh, this threshold ranges between USD 2462 and USD 7386.

**Results:**

The estimated ICERs were USD −99, USD −87, USD −4, USD 792, and USD 229 per QALY gained for healthcare personnel, older adults (≥60), children aged less than five years, adults with comorbid conditions, and pregnant women, respectively. For all risk groups, ICERs were below the WHO willingness-to-pay threshold for Bangladesh. Vaccinating pregnant women and adults with comorbid conditions was highly cost-effective per additional life year gained, while vaccinating healthcare personnel, older adults (≥60), and children under five years were cost-saving per additional life year gained.

**Conclusions:**

Influenza vaccination to all target risk groups in Bangladesh would be either cost-saving or cost-effective, per WHO guidelines of GDP-based thresholds.

Seasonal influenza causes significant mortality and morbidity globally, particularly in high-risk populations such as pregnant women, children under five years of age, adults with underlying health conditions, older adults (≥60), and healthcare personnel [1]. Influenza viruses cause an estimated one billion infections worldwide each year, with three to five million cases classified as severe; they also cause up to 650 000 deaths annually, largely in low- and middle-income countries (LMICs) [2,3]. Aside from direct health impact, seasonal influenza also causes significant healthcare costs, economic losses stemming from workdays missed due to influenza-associated illness, and decreased productivity [4].

Despite the recommendation from the Strategic Advisory Group of Experts on Immunization (SAGE) of the World Health Organization (WHO), the use of seasonal influenza vaccines among LMICs is low [1,5,6]. Seasonal influenza vaccination coverage in LMICs varies widely among high-risk populations, ranging from 2% to 72% among children, 10% to 70% among older adults (≥60), 0% to 4% among pregnant women, and 20% to 56% among healthcare personnel [5].

Among these groups, healthcare personnel require special attention in vaccination efforts, as they might be at a higher risk of infection due to occupational exposure during influenza epidemics [7]. Not only does their immunisation significantly curb the spread of influenza to vulnerable patients and the broader community, but it is also crucial in preserving vital healthcare services by reducing illness-related absence from work [8].

Despite the clear guidelines developed by the WHO, many countries have encountered challenges in implementing effective vaccination policies for high-risk populations. Addressing these obstacles and ensuring comprehensive vaccine coverage remains an ongoing struggle [9].

Influenza poses a significant burden in Bangladesh, with an estimated mortality rate of 6 per 100 000 for children under five years of age and 41 per 100 000 for persons aged >60 years [10]. Further, there are an estimated 4.4–6.7 influenza-associated hospitalisations and 100–170 outpatient visits for influenza-like illness (ILI) per 1000 population [11]. Further, around 25 million people of all ages seek outpatient treatment for influenza in Bangladesh, with an annual direct cost of USD 108 million, while approximately 30 592 people are hospitalised with laboratory-confirmed influenza each year, with an estimated cost of USD 1.4 million [11].

Bangladesh is one of the most densely populated countries globally, presenting an increased risk for the rapid spread of influenza during outbreaks. Its close-knit communities, paired with increased global connectivity and migration practices, make the country a potential hotspot for disease transmission. The interconnectedness of today’s world emphasises the urgent need for comprehensive preparedness and response measures to mitigate the impact of an influenza epidemic in Bangladesh.

Despite the heavy burden of influenza, Bangladesh, an LMCI, does not have a national influenza vaccination programme for high-risk populations because of insufficient evidence about its cost-effectiveness. Only Hajj pilgrims receive free vaccinations as a mandatory requirement by the Saudi Government [12]. The lack of robust contextual evidence on the economic benefits of vaccinations to the health care system and to society at large presents a challenge for promoting influenza vaccination policies [10,13-15].

While research from high-income and other LMICs settings suggests that influenza vaccination can be cost-effective, this may not be transferable to Bangladesh because of differences in health-seeking behaviour, health service delivery, and vaccination costs [13,14]. Context-specific estimates for Bangladesh could therefore help inform resource allocation decisions.

We performed a cost-effectiveness analysis on influenza vaccination in Bangladesh, focussing on high-risk groups, to gather evidence that would inform policy decisions on prioritising groups for influenza vaccination while ensuring the optimal allocation of limited resources for influenza prevention and control.

## METHODS

We performed a deterministic Markov decision-analytical model based on the guidelines of cost-effectiveness analyses (CEA) of health care programmes to recommend priority risk groups for seasonal influenza vaccination in Bangladesh [16]. We implemented the model for each of the five risk groups: pregnant women, children under five years of age, adults with underlying health conditions, older adults (≥60), and healthcare personnel. We chose a one-year time frame for intervention, assuming that influenza epidemics occur annually and that vaccination only provides protection for one season at a time [17]. We used a time horizon equal to each risk group's age-specific life expectancy after the age at which the first infection is reported [17]. To ensure incremental-cost-effectiveness ratios were consistent across all risk groups, we assumed a life expectancy of 75 years and the age of first infection for each risk group (children under five years: 5 years; health care personnel: 33 years; pregnant women: 18 years; adults with comorbidity condition: 45 years; older adults (≥60 years): 60 years). Thus, each of the risk groups had different life expectancy after first infection. Then, we assumed a discount rate of 5% to adjust the future costs and benefits associated with vaccination to their present value, taking into account the time preference for money. To carry out the economic evaluation, we estimated direct and indirect costs associated with ILI and severe acute respiratory illness (SARI), reflecting a societal perspective that includes direct and indirect costs [15], while also calculating the costs of productivity lost because of influenza-associated morbidity. We included all the input parameters in the model by examining the most probable values. Subsequently, using Monte Carlo simulations, we used a range of potential values around assumed probability distributions to investigate uncertainties and provide exhaustive data for decision-making.

### Data sources

#### Population

Following the WHO position paper for vaccines against influenza, we included five high-risk groups: pregnant women, children aged 6–59 months, older adults (≥60 years), healthcare personnel, and adults with chronic medical conditions. We compiled locally available data, both published and unpublished, to estimate the size of the population of each risk group [1]. Specifically, we obtained population sizes using the Bangladesh Statistical Yearbook 2020 and projected them through 2021 using Bangladesh’s population growth rate of 1% [18]. Using data from Bangladesh’s most recent published demographic and health census, we calculated the estimated number of pregnant women by multiplying the crude birth rate by the total number of women of childbearing age (15–49 years) [18,19]. We also estimated the number of adults with at least one chronic illness, including chronic obstructive pulmonary disease (COPD), asthma, chronic heart disease, hypertension, diabetes, and human immunodeficiency virus (HIV), using data from a Bangladesh household income and spending survey [20]. Lastly, we obtained population sizes of children under five years and older adults (≥60) from the Bangladesh Statistical Yearbook 2020 [18] and the number of healthcare personnel in Bangladesh from the Directorate General of Health Services web portal and a private health care institutions survey [21,22]. In Bangladesh, high-risk populations ranged from 199 779 registered healthcare personnel to 94 797 212 for adults with comorbidities.

#### Influenza-related health state transition probabilities

We used data from hospital-based influenza surveillance in Bangladesh to estimate risk group-specific influenza-associated attack rates. The surveillance programme has been ongoing since 2007 in nine tertiary care hospitals across Bangladesh and enrols >5000 SARI patients annually on average [23]. The surveillance tests oropharyngeal and nasopharyngeal swabs to confirm influenza. Influenza-related medical records, including outpatient visits and inpatient and emergency department records, were accessible in the surveillance metadata.

Using this data, we estimated risk-group-specific prevalence of ILI and influenza-associated hospitalisation rates for the 2020–21 influenza season. We considered deaths up to four weeks after hospital discharge as influenza-associated mortality resulting from SARI and ILI. We used the hospital-based influenza surveillance data for influenza-associated mortality rates. Where country-specific data were unavailable, we adjusted influenza attack rates using previously published data from neighbouring countries [10,23-38] (Table S1 in the **Online Supplementary Document**).

#### Vaccine coverage and effectiveness

A few studies conducted during 2008–09 quantified the effectiveness of the influenza vaccine among young children and pregnant women in Bangladesh, yet their estimates were imprecise [25]. Therefore, we adopted the WHO’s pooled vaccine effectiveness (VE) estimates for LMICs for the seasonal influenza vaccines in each risk group [5]. Table S1 in the **Online Supplementary Document** shows the VE and vaccine coverage used as input parameters for the model. The WHO systematic review reported risk group-specific vaccine effectiveness in various influenza disease outcomes, including ILI, laboratory-confirmed influenza, hospitalisation, and mortality [5]. Among these outcomes, we used the pooled VE for laboratory-confirmed influenza for each risk group and pooled vaccine coverage estimates for LMICs as input parameters for our model [5].

#### Costs related to medical and non-medical resource utilisation

We extracted risk group-specific direct medical and non-medical resource utilisation costs for ILI and SARI from a previously published icddrb study [11]. Here, medical resource utilisation costs included hospital registration fees, bed rental costs, medications, laboratory tests, and informal payments for influenza-related complications. The non-medical costs were those for food, caregiver’s lodging, and transportation costs for patients or their family members. These costs were categorised as out-of-pocket and hospital-supported costs. We used medical and non-medical costs to estimate the total direct cost per influenza episode (Table S2 in the **Online Supplementary Document**).

#### Productivity loss and indirect costs

We extracted the risk group-specific data on productivity loss using data from the aforementioned icddrb study in Bangladesh [11], which enrolled patients with laboratory-confirmed influenza infections from outpatient and inpatient departments of four tertiary care hospitals from May to October 2010. We estimated lost workdays by counting the number of days that caregivers missed due to taking care of sick personnel, and used a similar approach for case patients. We then computed monetary loss from the self-reported daily wage rates for lost workdays. For female caregivers who were homemakers and unemployed, we used the Government of Bangladesh's minimum salary rate of USD 77.54 per month to determine their productivity loss [39]. We excluded school absences for influenza-related illnesses from productivity loss calculations.

We then estimated the total indirect cost per illness for each case patient after calculating productivity loss separately for case patients and caregivers. After compiling these data, we calculated the indirect costs of outpatient and inpatient care (Table S2 in the **Online Supplementary Document**), excluding any indirect costs for individuals who did not seek treatment and died. All costs were assumed to be annual per person in local currency Bangladeshi taka (BDT).

#### Outcome measure

We used quality-adjusted life years (QALY) as the outcome of interest. As influenza-specific QALY scores for each risk group and for different levels of care in Bangladesh are not available, we relied on published measures to estimate potential levels of QALY [40,41]. Bilcke et al. [40] evaluated the burden of disease associated with ILI and clinically confirmed influenza using QALY scores for each level of treatment across all age groups. Chit et al. [41] reported levels of QALY by age group and incremental loss because of influenza. Here we combined their QALY measures to derive QALY measures by age group and level of care (not-seeking care, outpatient, inpatient, and death) (Table S3 in the **Online Supplementary Document**).

#### Decision analytic model

**Figure 1:**
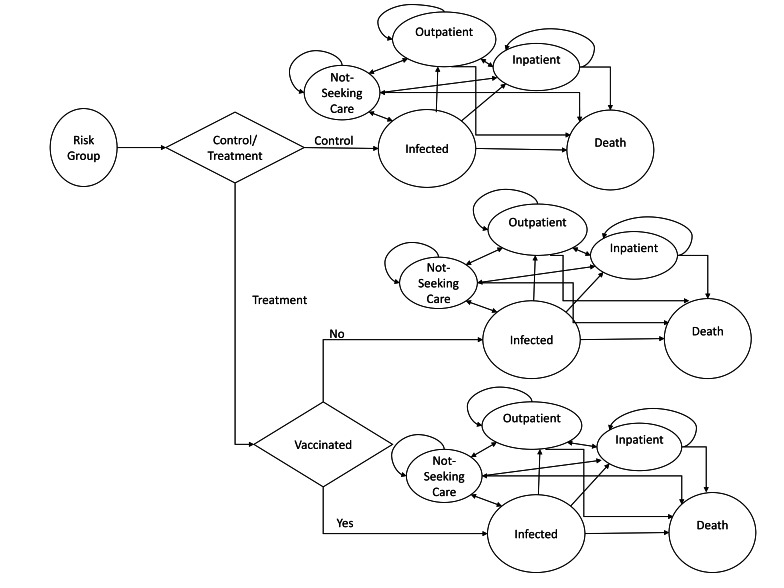
Markov decision-analytic model diagram for each risk group with four transition or health states. The arrows in the graph show the transition probabilities, representing the likelihood of transitioning between various states during a treatment cycle. Deaths were assumed to be in community or regional hospitals. All states were mutually exclusive, and death was an absorbing state, meaning those in the model who died could not later transition to any other state. We considered zero transition probabilities as the situation where backward transitions are not considered feasible in our model.

We developed a Markov decision analytic model (**Figure 1**) for each risk group, where each risk group was allocated to one of two arms: the unvaccinated and vaccinated arms. In the unvaccinated arm, each patient was assumed to be in one of four possible health states each year for the remainder of their life: community or not seeking care (initial state), outpatients, inpatients, and death. In the vaccinated arm, a percentage of the patients was assumed to be vaccinated and were expected to have lower levels of health service usage (outpatient and inpatient) and deaths (based on model inputs); those not vaccinated were expected to have the same levels of health service usage as those in the unvaccinated arm [42]. Deaths were assumed to be in community or regional hospitals. All states were mutually exclusive, and death was an absorbing state, meaning those in the model who died could not later transition to any other state. We considered zero transition probabilities as the situation where backward transitions are not considered feasible in our model (Table S1 in the **Online Supplementary Document**).

#### Estimating the incremental cost-effectiveness ratio

We calculated the incremental cost-effectiveness ratio (ICER) for each risk group to evaluate the cost-effectiveness of vaccination. To calculate this, we used the ratio of incremental cost to incremental QALY, where the increment was determined by comparing cost and QALY measures between the unvaccinated and vaccinated arms for respective risk groups. Incremental QALY between the unvaccinated and vaccinated population reflects gains in standardised life years (QALY) from vaccination. Meanwhile, incremental costs between the same comparison group reflect additional health system/societal costs of vaccination. Thus, the ratio of incremental cost to incremental QALY (ICER) reflects the additional cost for an additional QALY from vaccination. The estimated ICER can be compared across risk groups; the lower the ICER, the more cost-effective the intervention, with a negative ICER implying cost savings from an intervention. All costs were converted to 2021 BDT, according to annual inflation, and then converted to USD [43]. We considered a willingness-to-pay threshold (ICER) of less than one GDP per capita as highly cost-effective and of one to three times GDP per capita as cost-effective (per WHO guideline). For Bangladesh, this threshold ranges from USD 2462 to USD 7386 [43].

#### Sensitivity analyses

We performed a multivariable probabilistic sensitivity analysis using Monte Carlo simulations to address uncertainty in the model inputs. The key model’s parameters were changed simultaneously over predetermined probability distributions. We performed a Monte Carlo simulation with 1000 iterations with replacement to yield the distribution of parameters. All transition probabilities, QALYs, and vaccine effectiveness were assumed to follow the beta distribution, and the simulation randomly picked values around the mean assumed values. Note that the outpatient probability was assumed to have beta distribution, and each of the other three transition probabilities was assumed to be dependent on the respective weighted probability and simulated outpatient probability. This method ensured that the four transition probabilities were summed to 1 for each cycle of simulation. Similarly, outpatient, inpatient, and indirect cost measures were assumed to follow gamma distribution and simulation-selected random values based on the mean and standard deviation of assumed levels. Further, transition probabilities have a wide range across risk groups and health states, as seen from a ratio of maximum to minimum values for each probability (multiplier) (Table S4 in the **Online Supplementary Document**). Outpatient probability for children under five years ranged from a low of 5.4% to a high of 27.1% with an implied multiplier of 5, while older adults (≥60 years) had a range between 43.1% to 75.6% with an implied multiplier of 1.8. Other risk cohorts had multipliers within this range. We reported the mean as the primary results and the 5th and 95th percentiles as estimated confidence intervals around the mean (Table S4 in the **Online Supplementary Document**).

We also conducted a tornado analysis to assess the impact of assumed factors on the final outcome of interest, which is the ICER for each risk group [44,45]. Specifically, we used 18 input parameters for each risk group, encompassing three specific parameters (health state transition parameters, cost per day, and QALY) for each of the four stages of health. Additionally, we considered factors such as infection rate, vaccine uptake rates, vaccine prices, VE rates, and discount rates. We examined the ICER based on input factors whose values ranged from 90% to 110% of the relevant factor's mean. We performed all analyses in R, version 4.3 (R Core Team, Vienna, Austria).

## RESULTS

### Influenza illness and deaths averted by vaccination

The estimated annual number of outpatient cases averted by the seasonal influenza vaccine ranged from 3 720 833 for adults with comorbidities to 2717 for pregnant women. Older adults (≥60 years) had the largest number of annual hospitalisations averted due to seasonal influenza vaccination (n = 127 892), while pregnant women had the lowest (n = 1479). The annual averted number of influenza-associated deaths was expected to be 15 for health care personnel, 1160 for pregnant women, 15 738 for older adults (≥60 years), 5661 for adults with comorbidities, and 44 124 for children under five (**Table 1**).

**Table 1:** Clinical events and illness cost-averted by influenza vaccination and ICER by risk groups, Bangladesh

	Targeted high-risk groups				
	**Children under five years**	**Healthcare personnel**	**Adult with a comorbid condition**	**Pregnant women**	**Older adults (≥60 years)**
**Clinical events averted**					
Outpatient care	190 667	6771	3 720 833	2717	284 529
Inpatient care	53 767	2237	89 154	1479	127 892
Deaths	44 124	15	5661	1160	15 738
Cost averted by vaccination in USD*					
Direct cost					
*Outpatient care*	279 931	14 909	16 126 106	1 286	837 182
*Inpatient care*	497 424	51 796	4 348 107	86 225	2 757 427
Indirect cost	446 496	25 341	23 099 425	21 972	1 007 417
**ICER**					
Incremental cost in USD*	−17.010	−25.486	77.959	90.144	−31.265
Incremental QALY	3.523	0.244	0.154	0.399	0.349
ICER in USD (95% CI)*	−4.542 (−8.321, −0.764)	−99.047 (−130.158, −67.936)	792.566 (486.498, 1098.634)	229.035 (221.810, 236.260)	−87.405 (−105.041, −69.769)

CI – confidence interval, ICER – incremental cost-effectiveness ratio, QALY – quality-adjusted life years

*All cost was calculated by considering the annual inflation rate 6.10% and the dollar exchange rate of 109.00 BDT per USD.

### Vaccination cost

We found that the estimated annual direct vaccine programme cost was USD 222 272 for healthcare personnel; USD 175 million for adults with comorbid conditions; USD 11 million for children under the age of five, USD 2 million for pregnant women, and USD 6 million for older adults (≥60 years). As anticipated, healthcare personnel (who are generally healthier) had the lowest incremental health care cost compared to no vaccination, amounting to USD 51 796. Adults with comorbid conditions (who are likely less healthy) had the highest cost of USD 4 million. Likewise, the direct incremental health care cost was calculated at USD 777 355 for children under the age of five, USD 87 511 for pregnant mothers, USD 3 million for older adults (≥60 years), and USD 20 million for adults with comorbidities.

### ICER

**Figure 2:**
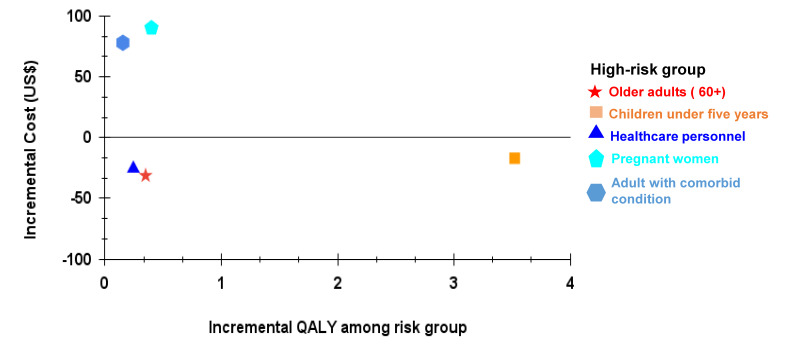
Cost-effectiveness plane showing incremental cost and QALY for each high-risk group to determine cost-effectiveness of vaccination. The figure shows that vaccinating healthcare personnel (incremental cost = −25.486, incremental QALY = 0.244), older adults (incremental cost = −31.265, incremental QALY = 0.349), and children under five years (incremental cost: −17.010, incremental QALY = 3.523) are cost saving, while vaccinating pregnant women (incremental cost = 90.144, incremental QALY = 0.399) and adults with comorbid conditions (incremental cost = 77.959, incremental QALY = 0.154) are cost effective based on WHO guidelines for cost-effectiveness threshold. Cost-effectiveness plane showing incremental cost and QALY for each high-risk group. Incremental cost for healthcare personnel was −25.486, while incremental QALY was 0.244, making the vaccination of this population cost-effective. Vaccinating older adults was cost-effective and less expensive since the incremental cost was −31.265 and the incremental QALY was 0.349. Vaccinating pregnant women was cost-effective and more expensive, with an additional cost of 90.144 and QALY of 0.399. For adults with comorbid conditions, incremental cost was 77.959 and incremental QALY was 0.154, indicating cost-effective and expensive vaccination. For children under five years, incremental cost was −17.010 and incremental QALY was 03.523, indicating vaccination was cost-effective and more expensive.

The estimated ICERs were USD −99, USD −87, USD −4, USD 792, and USD 229 per QALY gained for healthcare personnel, older adults (≥60 years), children under five years, adults with comorbid conditions, and pregnant women, respectively (**Table 1**). For all risk groups, ICERs were below the WHO willingness-to-pay threshold for Bangladesh and were thus cost-saving or cost-effective. Vaccinating healthcare personnel, older adults (≥60 years), and children under five years resulted in cost-saving per additional QALY. However, vaccinating pregnant women and adults with comorbid conditions was highly cost-effective per additional QALY gained, but not cost-saving (**Figure 2**).

### Sensitivity analysis

**Figure 3:**
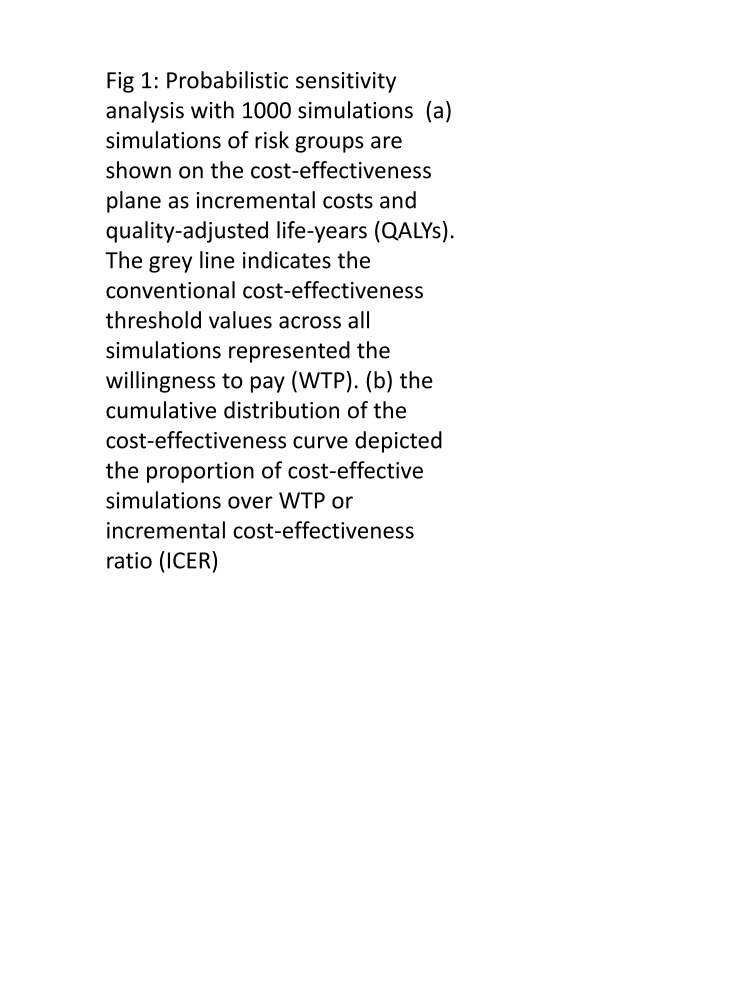
Probabilistic sensitivity analysis with 1000 simulations. The right-hand-side graphs display simulations of risk groups using incremental costs and QALYs on the cost-effectiveness plane. The grey line represents the conventional cost-effectiveness threshold values, which indicate the willingness-to-pay across all simulations. On the left-hand side, the graphs illustrate the cumulative distribution of the cost-effectiveness curve, showing the proportion of simulations that are considered cost-effective relative to the willingness-to-pay or ICER. **Panels A and B** show probabilistic sensitivity analysis (PSA) results for children under five years. **Panels C and D** show healthcare personnel. **Panels E and F** are related to adults with comorbid conditions. **Panels G and H** pregnant women. **Panels I and J** show the outcomes of the probability sensitivity analysis for older adults (≥60 years). Outpatient probability for children under five years ranged from a low of 5.4% to a high of 27.1% with an implied multiplier of 5, while older adults (≥60 years) had a range between 43.1% to 75.6% with an implied multiplier of 1.8. Other risk cohorts had multipliers within this range.

Our analysis showed that influenza vaccination of healthcare personnel, older adults (≥60 years), and children under five would be cost-saving (**Figure 3**). For these risk groups, vaccination would result in increased QALY, while the incremental cost would be lower, i.e. improved outcomes (QALY) from vaccination would be associated with reduced costs. In contrast, for pregnant women and adults with comorbidities, improved QALY from vaccination would be associated with increased costs. The cumulative distribution of the cost-effectiveness curve shows that as the willingness-to-pay increases, the percentage of simulations that are cost-effective increases. Thus, vaccinating all the risk groups against influenza is cost-effective, with the majority of ICER values being lower than threshold willingness-to-pay levels.

**Figure 4:**
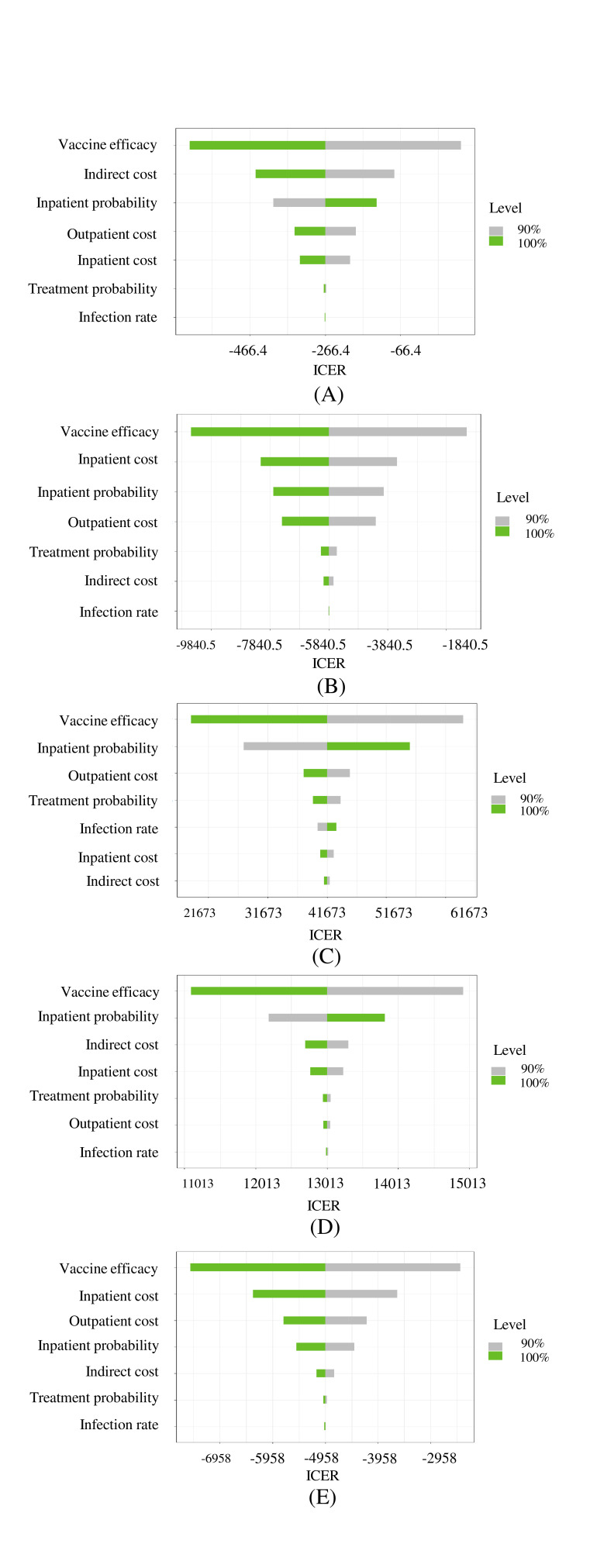
Tornado diagram for sensitivity analysis of incremental cost-effectiveness ratio for vaccinating each risk group. **Panel A.** Under five children: a low vaccine effectiveness (90% of the mean of vaccine effectiveness) implies a higher ICER, while a high vaccine effectiveness (110% of the mean vaccine effectiveness) implies a low ICER. Higher indirect cost also results in lower ICER. Higher inpatient probability results in higher ICER. **Panel B.** Healthcare personnel: among the seven factors, vaccine effectiveness appears to have the largest impact, indicating higher effectiveness results in lower ICER (or more cost-effectiveness). Higher inpatient cost also results in lower ICER. Higher outpatient cost also results in lower ICER. **Panel C.** Adults with comorbid conditions: among the seven factors, vaccine effectiveness appears to have the largest impact, indicating higher effectiveness results in lower ICER (or more cost-effectiveness). Higher inpatient probability results in higher ICER. **Panel D.** Pregnant women: among the seven factors, vaccine effectiveness appears to have the largest impact, indicating higher effectiveness results in lower ICER (or more cost-effectiveness). Higher inpatient probability results in higher ICER. **Panel E.** Older adults (≥60 years): among the seven factors, vaccine effectiveness appears to have the largest impact indicating higher effectiveness results in lower ICER (or more cost-effectiveness). It is important to note that higher inpatient probability results in lower ICER.

Our Tornado analysis showed that across all risk groups, increased vaccine effectiveness lowers ICER while lower inpatient probabilities (probability of being hospitalized due to influenza) can play a significant role in lowering ICER (**Figure 4**). For children under five, a low vaccine effectiveness (90% of the mean vaccine effectiveness) implied a higher ICER, while a high vaccine effectiveness (110% of the mean vaccine effectiveness) implied a low ICER. Higher inpatient probability resulted in higher ICER for children under five. Similarly, higher inpatient probabilities resulted in lower ICER for health care personnel and pregnant women. However, higher inpatient probabilities resulted in higher ICER for adults with comorbid conditions.

## DISCUSSION

We constructed a Markov decision-analytic model using available Bangladesh-specific data on influenza disease burden, including outpatient consultations, severe acute respiratory infection hospitalisations, deaths, and cost data to evaluate the cost-effectiveness of implementing an influenza vaccination programme for high-risk populations in Bangladesh. We observed that implementing such a programme for high-risk populations such as pregnant women and adults with underlying health conditions would be highly cost-effective and meet the guidelines suggested by WHO [46]. Additionally, vaccinating healthcare personnel, older adults (≥60 years), and children under five years would be cost-saving for Bangladesh. By investing in an influenza vaccination programme, Bangladesh could save lives, reduce health care costs associated with influenza illness, and improve health outcomes for vulnerable populations.

We found that vaccinating healthcare professionals, older adults (≥60 years), and children under five years would lead to the highest cost-savings among all target risk groups, as preventing influenza illness saved more money than the cost of the vaccination programme. Our static model did not account for potential additional benefits of vaccinating health care personnel, such as the prevention of nosocomial spread during seasonal epidemics. This means our assumption is conservative, as any potential indirect effects would likely enhance the cost-effectiveness of vaccinating this population.

Healthcare personnel in Bangladesh face a higher risk of contracting and spreading influenza, particularly in overcrowded hospital settings. Therefore, it is crucial to prioritise promoting influenza vaccination among this group to reduce nosocomial transmission during influenza epidemics. Although many countries, including LMICs, have implemented publicly funded influenza vaccination programmes for health care personnel, this is not the case with Bangladesh [9,17,47,48]. A meta-analysis by Ott et al. and cost-effectiveness analysis from the Lao People’s Democratic Republic support the cost-saving benefits of HCW vaccination programmes in similar settings to Bangladesh [17,49]. The Bangladesh government successfully vaccinates a significant number of Hajj pilgrims each year, totalling around 122 558 in 2023. This indicates that vaccinating a sizable cohort of patient-facing healthcare professionals, including doctors and nurses, in Bangladesh, which totals around 199 779, is feasible. Our analysis suggests that the investment needed for publicly funded vaccination of healthcare personnel is both feasible and potentially cost-saving for Bangladesh. It also provides evidence supporting the prioritisation of vaccinating the older adult (≥60 years) population in Bangladesh against influenza, as they are particularly susceptible to severe influenza outcomes due to compromised immune responses and the presence of comorbidities [37]. By prioritising vaccination among older individuals, Bangladesh could effectively mitigate the burden of influenza-related complications within this vulnerable population, safeguarding their health and well-being while alleviating strain on health care resources. Our findings reveal that vaccinating older adults (≥60 years) leads to economic gains, aligning with reports from other studies where the cost savings from preventing influenza complications outweigh the expenses of the vaccination programme [17,49].

We also found that vaccination of other high-risk groups, including pregnant women and adults with underlying health conditions, would be highly cost-effective and cost-saving for children under five. However, various factors should be considered if further prioritisation is needed due to limited resources, such as budgetary implications and existing platforms for vaccine delivery (e.g. the Expanded Program on Immunization and antenatal care for infants and pregnant women). Priority may also be given to those at higher risk of severe outcomes, such as individuals with specific chronic medical conditions. Ultimately, decision-makers should carefully weigh the potential impact and feasibility of vaccinating each target group to ensure optimal reduction of illness while considering logistical constraints and non-economic impacts.

Our sensitivity analysis highlighted the inpatient probability as one of the top three factors significantly influencing the ICER. Specifically, we observed that lower inpatient probability is associated with reduced ICER values, assuming other parameters like vaccine effectiveness remain constant. However, we suspect that our current input parameter here might be underestimated; if we were to use the true inpatient probability, it is likely that the ICER would increase, indicating the vaccine might be less cost-effective than reported here. Nonetheless, our sensitivity analysis also showed that even if we assume a higher inpatient probability than the current estimates, the ICER would still remain below the WHO threshold, i.e. would be considered cost-effective according to WHO guidelines.

Due to a significant correlation between outpatient and inpatient probability, we chose to exclude outpatient probability from the tornado analysis. However, our analysis indicates that the impact of outpatient probability on the ICER was opposite to that of inpatient probability. This means that a higher outpatient probability would result in a lower ICER, suggesting that the vaccine would be even more cost-effective than our current estimate, assuming that the current estimate of outpatient probabilities is underestimated.

Our study had several limitations. First, the resource utilisation and medical cost data came from surveys associated with SARI and ILI surveillance, not exclusively from laboratory-confirmed influenza cases. Hence, the cost data should be considered as proxies of resource utilisation and the medical cost of influenza illness in Bangladesh. Second, we did not consider the indirect effects of vaccination in our model, or more specifically, its impact on groups with a higher risk of spreading the infection to others, such as healthcare personnel, children under the age of five, and pregnant women. Hence, this is a conservative assumption, as any indirect effect would make vaccinating the target groups more cost-effective. Third, we observed some disparities among different risk groups in the model's input parameters in terms of outpatients and inpatient probabilities, likely due to the fact that the surveillance system, which captured patients from general medicine and paediatric wards, may have underrepresented number of inpatient visits for adults with comorbid conditions and pregnant women, as some of these patients may also have been admitted in other specialised units such as cardiology and obstetric departments. Fourthly, we relied on probabilities of deaths for healthcare personnel and pregnant women from other LMICs, as there was insufficient data available in Bangladesh. This could lead to either an underestimation or overestimation of the actual outcomes averted in these groups in our analysis. Similarly, we adopted QALY estimates from other contexts which may not be representative of Bangladesh. Finally, even though we address uncertainty in the result through robust Monte Carlo simulations with 1000 iterations and tornado analysis, uncertainty may still exist, which could be further reduced by using context-specific parameter distribution.

To address these limitations, future research should use robust input data to improve the appropriateness of model outputs and focus on developing a dynamic transmission model that considers both the direct and indirect effects of the vaccine on influenza transmission over time. Additionally, further studies should be conducted to estimate the cost of medical and non-medical resource utilisation in specific risk groups, particularly for laboratory-confirmed influenza cases. This would help optimise uncertainty in the model's assumptions and improve the accuracy of cost-effectiveness estimates.

## CONCLUSIONS

Our analysis demonstrates that introducing an influenza vaccination programme in Bangladesh that targets high-risk populations, including pregnant women and adults with underlying health conditions, would be highly cost-effective per WHO guidelines on GDP-based thresholds. Additionally, vaccinating healthcare personnel, older adults (≥60 years), and children under five years would be cost-saving to the Bangladeshi society and could be prioritised. Our analysis provides evidence to guide policy decisions and to advocate for the initiation of a publicly funded influenza vaccination programme targeting high-risk populations in Bangladesh.

## Additional material


Online Supplementary Document.
