# Supplementary material for: Cost-effectiveness of seasonal influenza vaccination in WHO-defined high-risk populations in Bangladesh
Source: J Glob Health. 2024 Jul 19;14:04126. doi: 10.7189/jogh.14.04126 (PMC11257706; doi:10.7189/jogh.14.04126)
Supplement: Online Supplementary Document. [file jogh-14-04126-s001.pdf]

Table S1: Input data for influenza epidemiology parameters stratified by targeted risk group

Parameters	Base-values	Source reference
**Influenza gross attack rate**		
Children under five years	0.1000	[1]
Healthcare personnel	0.1500	[2]
Adult with comorbid condition	0.3123	[3]
Pregnant women	0.1097	[4]
Older adults 60+	0.0400	[5]
**Probability of not seeking care**		
Children under five years	0.7818	Calculated*
Healthcare personnel	0.3755	Calculated*
Adult with comorbid condition	0.7133	Calculated*
Pregnant women	0.9690	Calculated*
Older adults 60+	0.1471	Calculated*
**Probability of outpatient**		
Children under five years	0.1450	[6]
Healthcare personnel	0.4680	[7]
Adult with comorbid condition	0.2800	[8]
Pregnant women	0.0153	[9]
Older adults 60+	0.5864	[10]
**Probability of inpatient**		
Children under five years	0.0402	Surveillance data
Healthcare personnel	0.1554	Surveillance data
Adult with comorbid condition	0.0063	Surveillance data
Pregnant women	0.0088	Surveillance data
Older adults 60+	0.2373	Surveillance data
**Probability of death**		
Children under five years	0.0329	[11]
Healthcare personnel	0.0010	[12]
Adult with comorbid condition	0.0004	[13]
Pregnant women	0.0069	[4]
Older adults 60+	0.0292	[11]
**Vaccine coverage of seasonal influenza vaccine**		
Children under five years	0.8000	[14]
Healthcare personnel	05600	[14]
Adult with comorbid condition	0.7200	[14]
Pregnant women	0.4900	[14]
Older adults 60+	0.7200	[14]
**Vaccine effectiveness**		
Children under five years	0.8100	[14]

Parameters	Base-values	Source reference
Healthcare personnel	0.5100	[14]
Adult with comorbid condition	0.6000	[14]
Pregnant women	0.5000	[14]
Older adults 60+	0.5800	[14]
**Vaccine cost, including per vaccine and vaccination cost (USD)**		
Children under five years	8.84	[15]
Healthcare personnel	9.85	[15]
Adult with comorbid condition	9.85	[15]
Pregnant women	9.85	[15]
Older adults 60+	9.85	[15]
*Probability of not seeking care= 1- probability (inpatient + outpatient + death)		

Table S2: Medical, non-medical resource utilization costs, indirect cost stratified by targeted risk group

Targeted risk group	Cost in each markov state (TK)			Source
	Indirect	Outpatient	Inpatient	
Children under five years	1,773	1,030	6,645	Survey data
Healthcare personnel	2,098	1,324	12,498	Survey data
Adult with comorbid condition	599	52	8,170	Survey data
Pregnant women	2,486	159	20,728	Survey data
Older adults 60+	1,658	1,361	12,780	Survey data

Table S3: Model input for QALY stratified by targeted risk group

Targeted risk group	Markov states				Source
	Not seeking care	Outpatient	Inpatient	Death	
**Children under five years**					
Base value	0.9200				[40,41]
Reduction in QALY	0.0146				[40,41]
Adjusted QALY	0.9100	0.8800	0.7900	0	[40,41]
**Pregnant women**					
Base value	0.8950				[40,41]
Reduction in QALY	0.0160				[40,41]
Adjusted QALY	0.8790	0.8500	0.7700	0	[40,41]
**Adult with comorbid condition**					

Targeted risk group	Markov states				Source
	Not seeking care	Outpatient	Inpatient	Death	
Base value	0.8500				[40,41]
Reduction in QALY	0.0170				[40,41]
Adjusted QALY	0.8330	0.8100	0.73000	0	[40,41]
**Healthcare personnel**					
Base value	0.9000				[40,41]
Reduction in QALY	0.0174				[40,41]
Adjusted QALY	0.8826	0.8600	0.7700	0	[40,41]
**Adults 60+**					
Base value	0.8500				[40,41]
Reduction in QALY	0.0170				[40,41]
Adjusted QALY	0.8330	0.8100	0.7300	0	[40,41]

Table S4: Probability sensitivity analysis (PSA) distribution

Parameters	Minimum	Maximum	Mean	Probability distribution in PSA
**Influenza gross attack rate**				
Children under five years	0.0256	0.2155	0.0998	Beta
Healthcare personnel	0.0733	0.2959	0.1513	Beta
Adult with a comorbid condition	0.1830	0.4415	0.3066	Beta
Pregnant women	0.0374	0.2169	0.1111	Beta
Older adults 60+	0.0252	0.2294	0.0996	Beta
**Probability of not seeking care**				
Children under five years	0.6657	0.8635	0.7801	Beta
Healthcare personnel	0.2682	0.4815	0.3754	Beta
Adult with a comorbid condition	0.5507	0.8510	0.7148	Beta
Pregnant women	0.9031	0.9840	0.9682	Beta
Older adults 60+	0.0674	0.1569	0.1133	Beta
**Probability of outpatient**				
Children under five years	0.0539	0.2706	0.1453	Beta
Healthcare personnel	0.3186	0.6205	0.4688	Beta
Adult with a comorbid condition	0.1405	0.4439	0.2781	Beta
Pregnant women	0.0001	0.0823	0.0161	Beta
Older adults 60+	0.4313	0.7558	0.5893	Beta
**Probability of inpatient**				
Children under five years	0.0350	0.0454	0.0410	Beta
Healthcare personnel	0.1106	0.1986	0.1548	Beta

Parameters	Minimum	Maximum	Mean	Probability distribution in PSA
Adult with comorbid condition	0.0051	0.0079	0.0067	Beta
Pregnant women	0.0082	0.0089	0.0088	Beta
Older adults 60+	0.1575	0.3667	0.2649	Beta
**Probability of death**				
Children under five years	0.0287	0.0372	0.0336	Beta
Healthcare personnel	0.0007	0.0013	0.0010	Beta
Adult with comorbid condition	0.0003	0.0005	0.0004	Beta
Pregnant women	0.0064	0.0070	0.0069	Beta
Older adults 60+	0.0194	0.0451	0.0326	Beta
**Vaccine coverage of seasonal influenza vaccine**				
Children under five years	0.0938	0.3334	0.1990	Beta
Healthcare personnel	0.2837	0.5816	0.4393	Beta
Adult with comorbid condition	0.1604	0.4376	0.2798	Beta
Pregnant women	0.3433	0.6523	0.5113	Beta
Older adults 60+	0.1569	0.4273	0.2801	Beta
**Vaccine effectiveness**				
Children under five years	0.6378	0.9164	0.8097	Beta
Healthcare personnel	0.3544	0.6629	0.5109	Beta
Adult with comorbid condition	0.4501	0.7515	0.6002	Beta
Pregnant women	0.3667	0.6468	0.5018	Beta
Older adults 60+	0.4261	0.7358	0.5768	Beta
**Outpatient cost (USD)**				
Children under five years	1.17	214.07	25.30	Gamma
Healthcare personnel	0.09	459.85	31.70	Gamma
Adult with comorbid condition	0.18	163.17	27.75	Gamma
Pregnant women	0.02	316.12	26.20	Gamma
Older adults 60+	0.06	211.38	27.80	Gamma
**Inpatient cost (USD)**				
Children under five years	3.06	348.95	76.61	Gamma
Healthcare personnel	3.28	641	140.10	Gamma
Adult with comorbid condition	0.70	655.86	109.00	Gamma
Pregnant women	0.02	1797.08	211.14	Gamma
Older adults 60+	0.24	566.14	110.13	Gamma
**Indirect cost (USD)**				
Children under five years	0.001	204.63	15.74	Gamma
Healthcare personnel	0.001	459.32	19.87	Gamma
Adult with comorbid condition	0.001	162.19	16.30	Gamma
Pregnant women	0.001	313.9	24.74	Gamma

Parameters	Minimum	Maximum	Mean	Probability distribution in PSA
Older adults 60+	0.001	169.33	15.09	Gamma
**QALY for not seeking care**				
Children under five years	0.8012	0.9737	0.9096	Gamma
Healthcare personnel	0.7449	0.9546	0.8809	Gamma
Adult with comorbid condition	0.7037	0.9401	0.8314	Gamma
Pregnant women	0.7706	0.9509	0.8802	Gamma
Older adults 60+	0.6648	0.9303	0.8309	Gamma
**QALY for outpatient care**				
Children under five years	0.6996	0.9809	0.8780	Gamma
Healthcare personnel	0.6871	0.9597	0.8602	Gamma
Adult with comorbid condition	0.6459	0.9419	0.8104	Gamma
Pregnant women	0.6687	0.9559	0.8502	Gamma
Older adults 60+	0.6016	0.9386	0.8089	Gamma
**QALY for inpatient care**				
Children under five years	0.6225	0.9091	0.7903	Gamma
Healthcare personnel	0.5965	0.8869	0.7694	Gamma
Adult with comorbid condition	0.5191	0.8793	0.7332	Gamma
Pregnant women	0.5639	0.8969	0.7705	Gamma
Older adults 60+	0.5405	0.8887	0.7329	Gamma
